# Assessing Cancer Signal during Oral Antiplatelet Therapy in the Food and Drug Administration Adverse Event Reporting System: Mission Impossible

**DOI:** 10.1055/s-0037-1615253

**Published:** 2018-01-29

**Authors:** Victor Serebruany, Moo Hyun Kim, Christian Thevathasan, Thomas Marciniak

**Affiliations:** 1 Division of Neurology, Johns Hopkins University, Baltimore, Maryland, United States; 2 Division of Cardiology, Dong-A University, Busan, South Korea; 3 University College London, London, United Kingdom; 4 Bethany Beach, Delaware, United States

**Keywords:** antiplatelet agents, cancer, safety, registry, FAERS

## Abstract

Whether aggressive prolonged dual antiplatelet therapy (DAPT) promotes solid cancer risks remains a critical unsolved issue. Since the evidence from randomized trials, affiliated U.S. Food and Drug Administration (FDA) reviews, meta-analyses, and national registries is mixed, the search is ongoing. The FDA Adverse Event Reporting System (FAERS) is a global passive surveillance repository requiring mandatory updates for serious events. We assessed the frequencies of co-reporting any cancers with oral antiplatelet agent (OAA) strategies in FAERS. We examined the entire FAERS database ( *n* = 8,604,889) with regard to monotherapy or DAPT with OAA, suspected causative role, and co-reporting any cancers ( *n* = 433,111). We extracted cancer cases during monotherapy with aspirin (20,984 out of 462,371 or 4.54%), clopidogrel (2,797 out of 62,791 or 4.45%), prasugrel (119 out of 4,364 or 2.73%), and ticagrelor (144 out of 8.268 or 1.71%). DAPT with clopidogrel reported (2,453 out of 58,101, or 4.22%); prasugrel (162 out of 4,036, or 4.01%); and ticagrelor (195 out of 5,302 or 3.68%) cancer reports all on top of aspirin. We conclude that FAERS is currently unreliable for adequate assessment of cancer risks during DAPT. The retrieved evidence appears random and sporadic, while associated cancers are heavily underreported or/and missed. Without stricter rules, better surveillance, and enforcements, oncology outcome research options in FAERS are challenging.

## Introduction

The link between optimal duration and content of dual antiplatelet therapy (DAPT) after percutaneous coronary interventions with associated cancer risks remains an unsolved critical medical issue. In fact, the role of antiplatelet agents in tumor growth and prognosis is not new, and currently under intense investigation. The potential mechanism(s) responsible for such a harmful association are currently under scrutiny; this includes easier metastatic dissemination due to instability of platelet–tumor cell aggregates and/or an inability to keep cancer cells locally in situ by exhausted platelets, 1 Moreover, the results of landmark DAPT trials revealed better vascular outcomes, but excess noncardiovascular deaths due to cancer for prolonged antiplatelet strategy. 2 Some randomized data and their analyses by the U.S. Food and Drug Administration (FDA) suggest that clopidogrel and prasugrel in DAPT, prasugrel in TRITON, ticagrelor in PEGASUS, vorapaxar in TRACER enhance cancer risks, while other data such as prasugrel in TRILOGY, ticagrelor in PLATO, and vorapaxar in TRA2P were negative. 3 4 5 6 7 Since the randomized evidence does not provide clear answers, analyzing large national registries to pick up a cancer signal may be helpful, as proven by the recent Korean HIRA database mining. 8

The FDA Adverse Event Reporting System (FAERS) is a database that contains information on adverse events and medication error reports submitted to the FDA. 9 Data mining algorithms have been developed for the quantitative detection of signals from this vast database, that is, a signal means and a statistical association between a drug and an adverse event. 10 Importantly, FAERS data are publicly available. 9 10 11 Thus, evidence from such a large, uniform, government-mandated repository may be helpful to identify “real-life” DAPT—co-reported cancers in the FAERS entirety.

## Methods

### Data Source

All FAERS reports including those originated in 2016 qualified, meaning that the initial report for the adverse event was dated in 2016, although the report can be received by the FDA (FDA Receipt Date) as late as fourth quarter of 2016. There may have been follow-up reports associated with the same case after 2016, but the initial report was generated earlier than 2017, and all repeated entries were disregarded. The pooled FAERS database was searched using the terms “aspirin,” “acetylsalicylic acid,” “clopidogrel,” “prasugrel,” “ticagrelor,” “Plavix,” “Iscover,” “Zyllt,” “Effient,” “Efient,” “Brilinta,” or “Brilique,” and any “cancer” which was reported as an adverse event. To avoid bias, data mining and statistics were performed by independent researchers at FDAble, LLC (Glastonbury, CT, www.fdable.com ), a for-profit group that specializes in FAERS database analyses.

### Outcomes

The primary endpoint of this study was the distribution of oral antiplatelet agents (OAAs) with or without co-reported cancers, with a cutoff in 2016, the latest year for which all the records for aspirin, clopidogrel, prasugrel, and ticagrelor have been updated in FAERS. To mitigate the issue of multiple reporting of a single event, originators were counted by unique case numbers rather than by report numbers. In lay terms, if a single case has three separate reports and each repeated report indicates different sources, the mandatory counting was a single first source, and the other upgrades were disregarded unless they added any previously missing information.

### Statistics

Analyses were done using Open VigilFDA v1.0.2, a web-based user interface for the FAERS database. This software allows for analysis of adverse drug events reported to the FDA. The reported adverse events could then be analyzed for “disproportionality” and scored using various measures of statistical significance. FAERS-reported cancers were compared among antiplatelet strategies. These contemporary statistical techniques compared the reported adverse events to expected adverse events, and allowed quantifying the additional risk/odds of the drug and adverse event to the general background noise. Proportional reporting ratios (PRR) and 95% confidence intervals were calculated as a measure of disproportionality of reporting, and PRRs were compared across antiplatelet regimens using Breslow-Day statistical methodology. Values above 1 suggest a disproportionate association of a drug and event. Roughly, values greater than 2 indicate that this drug to adverse event combination is twice as likely as all other combinations. Categorical variables were estimated among OAA using a chi-squared test, and continuous variables were compared using two-sample *t* -tests and nonparametric tests. Statistical analyses were performed using SPSS version 13 (Chicago, Illinois, United States).

## Results

A total of 8,604,889 reports were qualified. The majority ( *n* = 8,026,366) of reports contain no mention of OAAs, while 578,523 records contain reference to at least one of the classes, including 441,387 cases that reported aspirin use, followed by clopidogrel ( *n* = 115,642), ticagrelor ( *n* = 13,375), and prasugrel ( *n* = 8,119). The FAERS distribution of oral antiplatelet strategies and associated cancers is detailed in Table 1 . Among all single agent regimens, any cancer has been reported with aspirin monotherapy most frequently (4.54%), followed by clopidogrel (4.45%), prasugrel (2.73%), and finally ticagrelor (1.71%). With regard to any cancers reported on top of DAPT, the risks were somewhat similar among clopidogrel (4.22%), prasugrel (4.01%), and ticagrelor (3.68%). Alarmingly, some multi-OAA adverse events on top of aspirin made the list despite lack of any recommendations advocating for triple OAA.

**Table 1:** Statistical considerations for cancer and DAPT in FAERS

	Number of cases not reporting cancer	Number of cases reporting some form of cancer	Total number of cases	% of total cases reporting some form of cancer	Risk ratio	Risk ratio (95%CI)	*p* -Value	Comment
Clopidogrel (no aspirin)	59,994	2,797	62,791	4.45	1.0551	(1.0006–1.1125)	0.04743	Cancer reported more frequently w/ clopidogrel alone (compared with clopidogrel + aspirin)
Clopidogrel + aspirin	55,648	2,453	58,101	4.22				
Prasugrel (no aspirin)	4,245	119	4,364	2.73	0.6794	(0.5383–0.8574)	0.00105	Cancer reported more frequently w/ prasugrel + aspirin (compared with prasugrel alone)
Prasugrel + aspirin	3,874	162	4,036	4.01				
Ticagrelor (no aspirin)	8,268	144	8,412	1.71	0.4654	(0.3763–0.5757)	< 0.0001	Cancer reported more frequently w/ ticagrelor + aspirin (compared with ticagrelor alone)
Ticagrelor + aspirin	5,107	195	5,302	3.68				
Aspirin (no clopidogrel, no prasugrel, no ticagrelor)	441,387	20,984	462,371	4.54	1.0749	(1.0318–1.1198)	0.00053	Cancer reported more frequently w/ aspirin alone (compared with aspirin + clopidogrel)
Clopidogrel + aspirin	55,648	2,453	58,101	4.22				
Aspirin (no clopidogrel, no prasugrel, no ticagrelor)	441,387	20,984	462,371	4.54	1.1307	(0.9718–1.3155)	0.111	Cancer reported more frequently w/ aspirin alone (compared with aspirin + prasugrel)
Prasugrel + aspirin	3,874	162	4,036	4.01				
Aspirin (no clopidogrel, no prasugrel, no ticagrelor)	441,387	20,984	462,371	4.54	1.234	(1.0745–1.4171)	0.00273	Cancer reported more frequently w/ aspirin alone (compared with aspirin + ticagrelor)
Ticagrelor + aspirin	5,107	195	5,302	3.68				
Clopidogrel (no aspirin)	59,994	2,797	62,791	4.45	1.6336	(1.3633–1.9574)	< 0.0001	Cancer reported more frequently for clopidogrel (no aspirin) compared with prasugrel (no aspirin)
Prasugrel (no aspirin)	4,245	119	4,364	2.73				
Clopidogrel (no aspirin)	59,994	2,797	62,791	4.45	2.6021	(2.2043–3.0718)	< 0.0001	Cancer reported more frequently for clopidogrel (no aspirin) compared with ticagrelor (no aspirin)
Ticagrelor (no aspirin)	8,268	144	8,412	1.71				
Prasugrel (no aspirin)	4,245	119	4,364	2.73	1.5929	(1.253–2.0251)	0.00013	Cancer reported more frequently for prasugrel (no aspirin) compared with ticagrelor (no aspirin)
Ticagrelor (no aspirin)	8,268	144	8,412	1.71				

## Discussion

Data from this large, uniform U.S. government-run international registry revealed that FAERS in the present form is not wholly suitable to adequately assess and distinguish cancer risks during modern antiplatelet strategies. It seems that better FAERS monitoring implying stricter rules and enforcements is warranted. Our data indicate potential massive cancer underreporting during DAPT, and missed entries within the entire spectrum of originated reports. These shortcomings challenge quality of outcome research and establishing drug interactions with adverse events by applying FAERS data. The index data are in agreement with our previous experience with FAERS confirming poor quality of event reporting. 12 13 Indeed, the missing data are well recognized as a major limitation of FAERS. 14 15

Why we are so sure that many reports were incomplete, or missing? Just a simple glance through the data on prasugrel and ticagrelor in Table 1 clearly indicated the problem with both newer antiplatelet agents which should be mandatorily used on top of aspirin. There is no single indication, or any international recommendation advocating monotherapy with ticagrelor or prasugrel. Therefore, if the assessment of cancer risks in FAERS was valid, both antiplatelet agents should yield similar rates for monotherapy versus DAPT-affiliated risks. Keeping in mind that any FAERS-reported “monotherapy” with prasugrel or ticagrelor is in fact an error for missed aspirin entries, then the rates of cancer with and without aspirin should be similar. However, DAPT with prasugrel and ticagrelor yielded much higher cancer rates, suggesting better quality of reporting rather than a real scientific finding. In short, reports of prasugrel and/or ticagrelor “without” aspirin are more “sloppy,” than those indicative of DAPT, which pick up cancer signals more frequently. Another observation in Table 1 is the finding that the highest cancer risks are observed after monotherapy with aspirin and clopidogrel. These data contradict all available randomized evidence and FDA reviews, and is probably attributed to the different reporting patterns of generic versus branded medications. In fact, sponsors of patented antiplatelet agents are well-aware of potential cancer risks observed in the indication-seeking trials and FDA secondary reviews. Therefore, drug manufacturers may be more “creative” in avoiding cancer reports affiliated with their branded agents. That is concerning, especially since direct reports to FAERS are rare, and more than 98% of cases are filtered by pharmaceutical companies. 13

There are few other important considerations which may be yielded from the index data. It seems that the quality of FAERS reports was similarly average for all antiplatelet agents. There is nothing unique about any particular drug reporting quality, and all data are suffering from missing entries. Massive missed or/and unknown cancer statuses preclude from better understanding of the drugs' safety profiles somewhat challenges the entire idea behind FAERS. Since the FDA mandates and oversees this valuable huge repository keeping it public, improving the quality of reports should be the utmost priority. In fairness, cancer reporting may be tricky, as there are numerous reasons for not complying with the FAERS mandated laws. Different patterns of cancer detection and nonuniform oncological diagnostic classifications around the globe may be partially responsible for the filing failures. Moreover, most cancer types in FAERS, especially as secondary noncausative measures, are heavily missing. Nevertheless, this study has important practical implications. First and foremost, the quality of the FAERS cancer reporting is unacceptable, raising concerns beyond antiplatelet agent analysis. Considering that U.S. filing is considerably better than report quality around the globe, 13 it seems as though the FDA should consider better options to stimulate proper international reporting, potentially switching such responsibility to the consumers, or health care professionals and away from manufacturers. Acknowledging sharp declines in ongoing or planned clinical trials with antiplatelet agents, the “real-life” data from FAERS is definitely useful if properly managed. Since FAERS entries are public, any scientist may access the data and our own within this huge repository. Moreover, FAERS maintenance is paid by U.S. tax dollars, requiring formal optimization and better surveillance.
